# High efficiency quantum cascade laser frequency comb

**DOI:** 10.1038/srep43806

**Published:** 2017-03-06

**Authors:** Quanyong Lu, Donghai Wu, Steven Slivken, Manijeh Razeghi

**Affiliations:** 1 Center for Quantum Devices, Department of Electrical Engineering and Computer Science, Northwestern University, Evanston, IL 60208, USA

## Abstract

An efficient mid-infrared frequency comb source is of great interest to high speed, high resolution spectroscopy and metrology. Here we demonstrate a mid-IR quantum cascade laser frequency comb with a high power output and narrow beatnote linewidth at room temperature. The active region was designed with a strong-coupling between the injector and the upper lasing level for high internal quantum efficiency and a broadband gain. The group velocity dispersion was engineered for efficient, broadband mode-locking via four wave mixing. The comb device exhibits a narrow intermode beatnote linewidth of 50.5 Hz and a maximum wall-plug efficiency of 6.5% covering a spectral coverage of 110 cm^−1^ at λ ~ 8 μm. The efficiency is improved by a factor of 6 compared with previous demonstrations. The high power efficiency and narrow beatnote linewidth will greatly expand the applications of quantum cascade laser frequency combs including high-precision remote sensing and spectroscopy.

Optical frequency combs^1^ emitting a broad spectrum of discrete, evenly spaced narrow lines with well-defined phase, have become attractive laser sources for a variety of applications. Particularly, they provide a unique combination of large wavelength coverage and high spectral resolution, therefore they allow for simultaneous, precise, and rapid spectroscopy of wide wavelength regions of interest^2^,^3^. This is of great importance to the mid-infrared (mid-IR) wavelength regime in which many strong fundamental ro-vibrational molecular transitions take place. High power frequency comb sources in the mid-IR range will revolutionize the trace gas analysis in environmental or medical monitoring applications^4^.

Mode-locked lasers^5^ can directly emit frequency comb output and have been the dominant comb sources in the near-infrared (near-IR) spectral region. However, there are few comb counterparts in the mid-IR range. Difference frequency generation (DFG)^6^ has been able to transform the near-IR frequency comb into the mid-IR range by mixing it with a continuous wave laser inside a nonlinear crystal, with nW power level per mode tooth^7^,^8^. The power was further boosted up to μW level by using an optical parametric oscillator^9^ technique which provides optical gain via parametric amplification^10^. Microresonator-based frequency combs^11^ have been able to produce mW power per mode in the mid-IR range by taking advantages of the ultrahigh-Q microresonator designs and powerful pumping sources^12^. Nevertheless, all of these technique requires external pumping sources and expensive optical components.

Quantum cascade laser (QCL) frequency combs, on the other hand, have been demonstrated as promising semiconductor frequency comb sources in the mid-IR and THz realm in the past few years^13^,^14^,^15^,^16^. Since the frequency comb is directly generated inside the QCL without any extra optical components, QCL frequency combs are monolithic and chip-based comb sources offering great promise for high-speed, high-resolution spectroscopy. Mid-IR and THz spectroscopy experiments using QCL frequency combs have also been recently demonstrated with high precision and low noise^17^,^18^. Higher power and uniform power distribution among the comb modes will always benefit the applications with less scanning time and higher sensitivity. Currently, a demonstrated QCL frequency comb at λ ~ 9 μm emits an average power-per-mode of about 0.6 mW per mode at room temperature (20 °C)^15^. The uniformity of power distribution is further improved via Gires–Tournois interferometers (GTI)-coated QCL combs, but the average power on each mode remains the same, about 0.5 mW at −6 °C^19^.

## Results and Discussion

Here we report high power room temperature QCL frequency combs at λ ~ 8.0 μm. A highly efficient strain-balanced active region was designed with broadband gain and low dispersion. The QCL frequency comb emits a very narrow intermode beating linewidth of 50.5 Hz and a high wall-plug efficiency (WPE) of 6.5% with a broad spectral coverage of 110 cm^−1^ for 290 modes and a significantly improved average power-per-mode distribution of ~3 mW.

**Figure 1:**
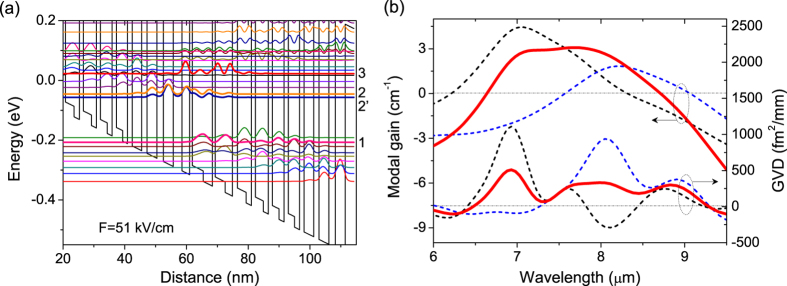
(**a**) Band structure of a strain-balanced active region designed at λ ~ 8.5 μm for frequency comb. The layer sequence in nm, starting from the injection barrier, is **2.8**/ 2.5/ **0.9**/ *3.5*/ 3.0/ **0.9**/ *3.2*/ 2.4/ **1.6**/ *2.8*/ 1.7/ **1.3**/ *2.6*/ 1.6/ **1.3**/ *2.2*/ 1.6/ **1.4**/ *2.0*/ 1.6/ **1.5**/ 3.1/ **1.7**/ 3.2/ **2.1**/ 3.2. The barriers are in bold font, and the wells are in normal font, the Ga_0.47_In_0.53_As insertions are in italic, and the underlined layers are doped to *n* = 1.7 × 10^17^ cm^−3^. The layer sequence for the design at λ ~ 7.5 μm is similar to ref. 20 with the injection barrier increased by 0.2 nm. (**b**) Calculated gain and GVD spectra for the single-core design (dashed lines) and dual-core design (solid lines) at a current density of 2.0 kA/cm^2^.

In this work, the QCL structure is based on a dual-core active region structure with a strong-coupled strain-balanced emitter designs at λ ~ 7.5 and 8.5 μm. Figure 1(a) is the band structure of the longer-wavelength active region design at λ ~ 8.5 μm. A strong coupling between the upper lasing level 2 and injector ground level 2′ was engineered by using a relatively thin injection barrier, which increases not only the quantum efficiency of the laser, but also increase the gain bandwidth^20^. Each structure was designed with a similar upper level lifetime of ~0.6 ps and was engineered to minimize cross-absorption between the two cores. Given the same doping concentration of 2.5 × 10^16^ cm^−3^, the same maximum current density is expected for each active core. This is important to obtain a flat-top gain and uniform power distribution for frequency comb operation. Figure 1(b) is the simulated gain spectrum calculated at a current density of 2.0 kA/cm^2^ following the description in ref. 20. The contribution of the gain from each active core is adjusted according to their optical confinement factor within the laser waveguide. The dispersions induced by gains from single cores and dual cores are calculated through the Kramers-Kronig relation, as plotted in Fig. 1(b). Between wavelengths of 7.5 and 8.5 μm, group velocity dispersions (GVDs) induced by dual-core design less than 350 fs^2^/mm are achieved. On account of the QCL material dispersion of −840 fs^2^/mm and waveguide dispersion of 1000 fs^2^/mm at λ ~ 8.0 μm, a total GVD 510 of fs^2^/mm is estimated, which is sufficiently low for mode locking of dispersed cavity modes into evenly spaced comb modes via four-wave mixing.

**Figure 2:**
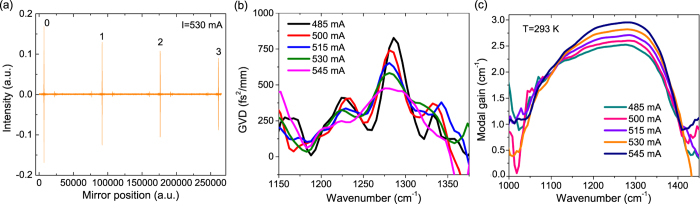
(**a**) Interferogram generated by a FTIR when measuring the subthreshold spectrum of a HR-coated comb device. Individual bursts in the interferogram are labelled sequentially. The resolution is 0.11 cm^−1^. (**b**) Measurement of the GVD and (**c**) gain of HR-coated QCL-comb as a function of current.

A Fourier transform technique was first performed to evaluate the amount of residual GVD inside a 4 mm-long high-reflection (HR) QCL frequency comb^21^ (See Method section). The time domain interferogram taken by the Fourier transform infrared (FTIR) is essentially the Fourier transform of the spectrum in the frequency domain. Due to the facet reflectivity, the sub-threshold spontaneous emission exhibits resonant cavity effects, resulting in “bursts” in the interferogram (Fig. 2(a)). Burst 1, as labelled in Fig. 2(a), corresponds to the moving mirror position at which the interferometer optical path difference matches a single round trip optical distance within the laser cavity. The Fourier transform of this burst will represent the phase spectrum and amplified spontaneous emission (ASE) spectrum in the cavity. The GVD, defined by the group delay dispersion per unit length, are presented in Fig. 2(b) by performing the second derivative of the relative phase divided the total travelling distance inside the cavity. Clearly, the HR-coated comb device exhibits net positive GVDs in the lasing spectral range. As the currents increases near threshold, the net GVD decreases to ~460 fs^2^/mm at 1270 cm^−1^, which is sufficient low for the modes to be efficiently coupled by four-wave mixing^15^,^22^. In addition, the modal gain is calculated by using the ASE spectra transformed from burst 1 and 3, as shown in Fig. 2(c). A broad flat-top gain with full width at half maximum (FWHM) of 350 cm^−1^ also verifies the above broad gain active design.

**Figure 3:**
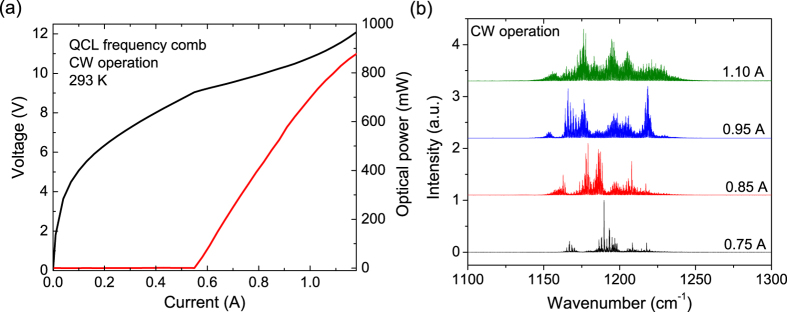
(**a**) *P-I-V* characterization of a 4-mm long HR coated QCL comb device in CW operation at 293 K. (**b**) Lasing spectra at different currents changing from 0.75 to 1.1 A. The resolution is 0.125 cm^−1^.

**Figure 4:**
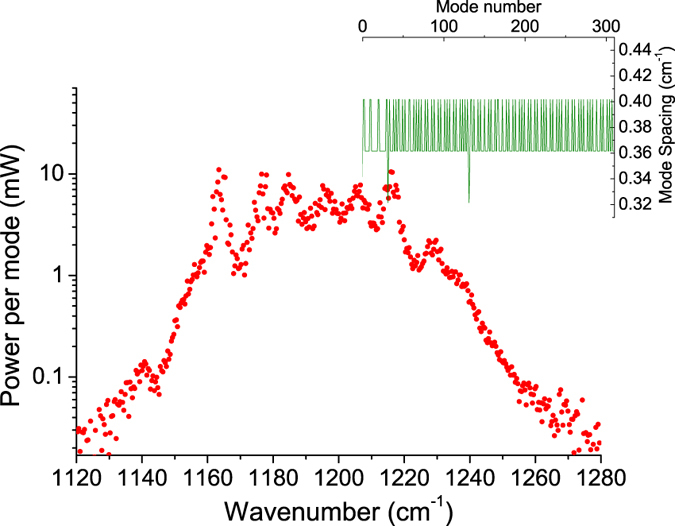
Power-per-mode distribution at a current of 1.06 A. The inset is the intermode spacing among different modes.

High CW power operation at room temperature was obtained for this frequency comb device as the optical power-current-voltage (*P*-*I*-*V*) characterization shown in Fig. 3(a). This device emits a CW power up to 880 mW with a threshold current density of 2.05 kA/cm^2^. In pulsed mode operation, the device emits up to 1.7 W with a threshold current density of 1.76 kA/cm^2^. The slope efficiency and WPE are 2 W/A and 10.3% in pulsed mode operation, and 1.7 W/A and 6.5% in CW operation at room temperature. This is compared with the WPEs less than 1% from previous QCL frequency comb demonstrations^13^,^15^,^19^. The spectra measurements were performed on a Bruker FTIR spectrometer with a liquid nitrogen cooled mercury-cadmium-telluride (MCT) detector in rapid scan mode. The emitting spectra shown in Fig. 3(b) reveal that the device operates in single mode in the lower current range, and exhibits a broad lasing spectra at the higher currents with a coverage up to 110 cm^−1^. The power-per-mode distribution at a current of 1.06 A is plotted on a logarithmic scale to further assess the uniformity of the emission, as shown in Fig. 4. The power distribution is much more uniform that the previous demonstration^9^, with over 1 mW power for 77% of all the modes, and a high average power-per-mode of about 3 mW. The intermode spacings among the 290 frequency comb modes are rather constant, ~0.38 cm^−1^.

**Figure 5:**
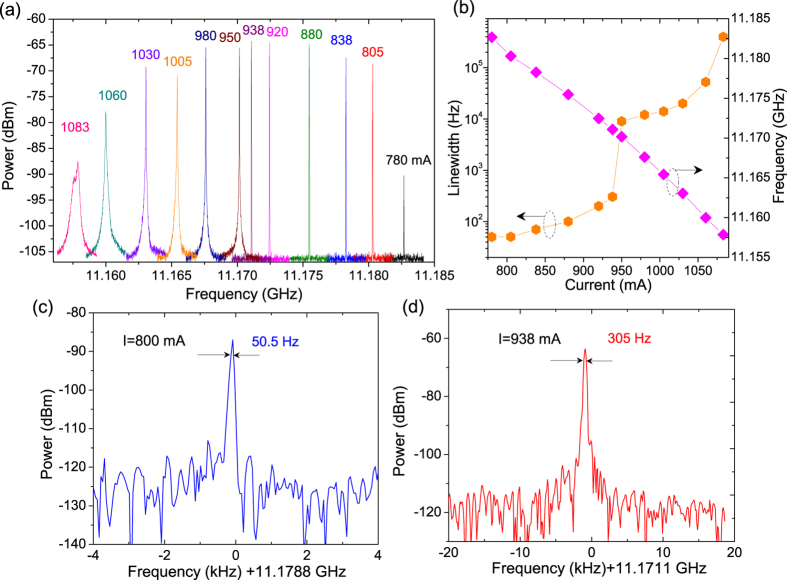
(**a**) Beatnote spectra at different currents at 293 K. (**b**) Beatnote linewidth and frequency as functions of currents. Beatnote spectra at currents of (**c**) 800 mA and (**d**) 938 mA at 293 K.

To assess frequency comb operation via phase locking of adjacent laser modes, the linewidth of the intermode beating was measured at the round-trip frequency with a high-speed quantum well infrared detector (QWIP) and a spectrum analyzer (Agilent-E4407B). A high stability current driver (Wavelength Electronics QCL2000 LAB) is used for low-noise testing. Figure 5(a) plots the beatnote spectra at different currents. The spectra were acquired using a spectrum analyzer with a span range of 3 MHz and resolution bandwidth (RBW) of 10 kHz. The beatnote spectra stay fairly narrow in the current range of 780–938 mA with a full width at half maximum (FWHM) is limited by the RBW. To further evaluate the linewidth, high resolution scans were performed with a RBW of 30 Hz and span range of 100 kHz. Extremely narrow beatnote linewidths of 50.5 at 11.18096 GHz and 305 Hz at 11.1711 GHz were observed at currents of 800 and 938 mA, respectively (Fig. 5(c and d)). The corresponding powers and spectral ranges are 400 mW and 610 mW, and 75 cm^−1^ and 95 cm^−1^, respectively. This narrow linewidth reflects that a well-defined phase is established among the frequency comb modes. A closer look of the beatnote linewidth and frequency as functions of currents is presented in Fig. 5(b). The frequency comb dynamic current range *ΔI*_*f*_ over the entire laser emitting range from the threshold to the roll-over currents *ΔI* is estimated to be 25%, which is much wider than the previous demonstrations with about 10% comb dynamic range^13^,^15^. At higher currents above 940 mA, the FWHM of the beatnote spectrum increases from 15 kHz at 950 mA to 52.5 kHz at 1060 mA with an output power of 770 mW. After the near roll over current of 1083 mA, the beatnote spectrum broadens to 400 kHz with a flat top, which indicates the device operates in the high-phase-noise regime^19^. The intermode beat frequency shifts as a function of current, with a tuning rate of 70 kHz/mA. This is because both the repetition frequency *f*_*rep*_ and the carrier-envelope offset frequency *f*_*ceo*_ are related to the group index *n*_*g*_, and are sensitive to the current induced temperature change. To realize a “ruggedized” broadband frequency comb for real-world applications, the comb spectrum and power will be stabilized by phase-locking to a reference stabilized frequency comb source^23^ or adjusting the operating temperature accordingly to stabilize the frequency comb by using the feedback of beatnote frequency change.

**Figure 6:**
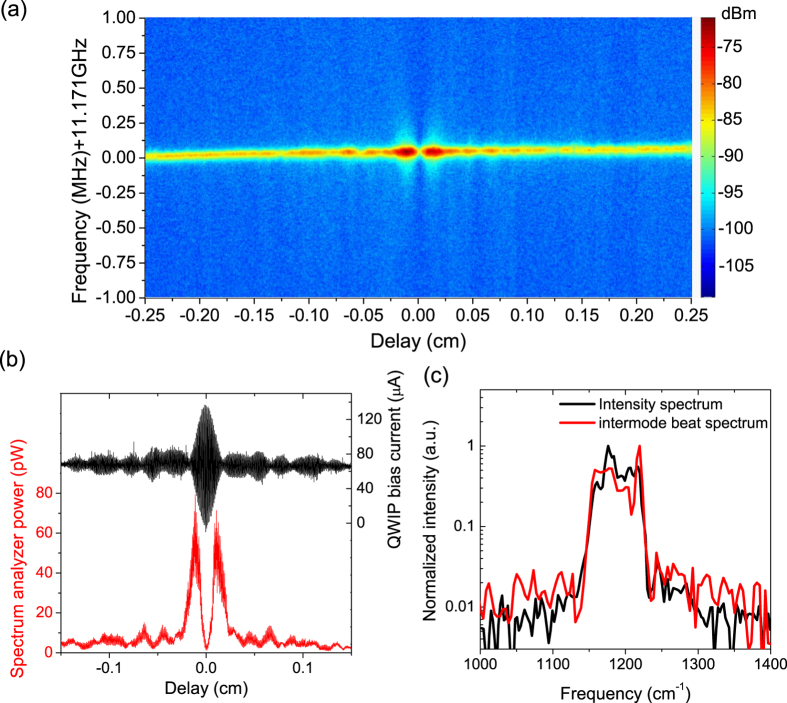
(**a**) Intermode beat spectroscopy taken at 938 mA. (**b**) Intermode beat interferogram (red) and intensity interferogram (black) measured with intermode beat spectroscopy. (**c**) Fourier transformed intermode beat spectrum (red) and intensity spectrum (black).

An intermode beat spectroscopy was performed to examine whether all the laser modes participate in the comb operation and gain insight in to the respective phase and coherent properties of the comb modes^13^,^15^. After the laser light was guided into the Michelson interferometer of the FTIR and refocused into the QWIP, beatnote spectra were recorded for each mirror position during a step scan. Figure 6(a) shows the beatnote interferogram at a current of 938 mA acquired with a resolution bandwidth of 30 kHz, a scan range of 2 MHz and a step scan resolution 4 cm^−1^. The peak beatnote powers measured by the spectrum analyzer and biased QWIP currents at different mirror positions were plotted together in Fig. 6(b). Like the previous demonstrations, a minimum of the intermode beat interferogram is observed near the zero time delay position, indicating a well-defined phase relation between the modes. The Fourier-transformed intermode beat spectrum is almost identical to the intensity spectrum, as shown in Fig. 6(c). Nearly the entire lasing spectrum with spectral bandwidth of 95 cm^−1^ contributes to the intermode beating and frequency comb formation. To further increase the spectral bandwidth even up to the octave range for mid-IR frequency comb sources, a broader gain design based on a balanced-gain heterogeneous active structure^24^ with a double-chirped mirror for dispersion engineering^14^ will be investigated in the next research stage.

## Conclusions

In conclusion, we demonstrate a frequency comb source based on a dispersion-compensated quantum cascade laser frequency comb at λ ~ 8 μm with high power output up to 880 mW for ~290 modes, covering a spectral coverage of 110 cm^−1^. The wall-plug efficiency is 6.5%, enhanced by a factor of 6 compared with the previous results. Extremely narrow beatnote linewidths less than 305 Hz is identified over a wide current range of 25% of total laser dynamic current range. The demonstrated monolithic high power efficiency frequency comb source will find wide applications especially in remote spectroscopy and sensing where high power output is mostly desired.

## Methods

### Growth and fabrication

The QCL structure presented in this work is based on the strain-balanced Al_0.63_In_0.37_As/Ga_0.35_In_0.65_As/Ga_0.47_In_0.53_As material system grown by gas-source molecular beam epitaxy (MBE) on an n-InP substrate. The growth started with a 2-μm InP buffer layer (Si, ~2 × 10^16^ cm^−3^). The laser core consisted of a dual-core strain-balanced single-phonon resonance (SPR) structure, which 20 stages for each wavelength design. The average doping of the active region is ~2.5 × 10^16^ cm^−3^. The MBE growth ended with a 30 nm-thick InP cladding layer (Si, ~2 × 10^16^ cm^−3^). Metal organic chemical vapor phase deposition (MOCVD) was then used for the growth of a 4.5-μm-thick InP cladding layer (Si, ~2–5 × 10^16^ cm^−3^) and 0.5-μm-thick InP cap layer (Si, ~5 × 10^18^ cm^−3^).

The wafer was processed into a standard buried ridge geometry with a ridge width of 7 μm. A 4-mm long QCL frequency comb device was high-reflection coated with Y_2_O_3_/Au (500/100 nm) and epi-down mounted on a diamond submount for characterizations. Testing was done on a thermoelectric cooler (TEC) stage at room temperature. For continuous wave (CW) measurement, the optical power was measured with a calibrated thermopile detector placed directly in front of the laser facet.

### Gain and GVD measurement

The gain and GVD spectra are acquired by using a Fourier transform technique^21^. The characterization is done by measuring the spontaneous emission of the QCL device under subthreshold CW operation with a FTIR. The emitted beam is splitted into two by the FTIR beam-splitter and reflected back by two mirrors and recombined into a MCT detector. The electrical fields for the two beams are expressed as:


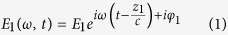



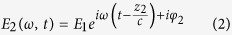


Here *z*_1_ and *z*_2_ are beam travelling distances, and *φ*_1_ and *φ*_2_ are their phases. *c* is the light speed in vacuum. Hence the total intensities of the two light beams detected by the MCT detector is:


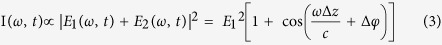


Here the Δz/*c* is the time delay between the two beams, Δ*φ* is their relative phase. Fourier transform of equation (3) for the burst 1 labelled in Fig. 2(a) will generate ASE spectrum and the relative phase spectrum after a single round-trip travelling inside the cavity. The GVD is therefore deduced by performing the second derivative of the relative phase divided the round-trip travelling distance *D*:


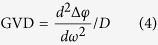


The ratio of two adjacent ASE spectra follows the relation^25^:


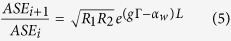


where *g*Г is the modal gain, *R*_1_ and *R*_2_ are facet reflectivities, *α*_*w*_ is waveguide loss, and *L* is cavity length. Therefore, the modal gain is calculated by using the ASE spectra transformed from burst 1 and 3 to average out some of the noise:


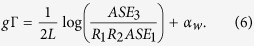


## Additional Information

**How to cite this article:** Lu, Q. *et al*. High efficiency quantum cascade laser frequency comb. *Sci. Rep.* **7**, 43806; doi: 10.1038/srep43806 (2017).

**Publisher's note:** Springer Nature remains neutral with regard to jurisdictional claims in published maps and institutional affiliations.
